# The Durability of Anti-TNF Therapy for Crohn’s Disease Is Higher in Anti-TNF Naïve Patients and Increases With Proactive Therapeutic Drug Monitoring

**DOI:** 10.1093/crocol/otaf028

**Published:** 2025-04-09

**Authors:** Robert Gilmore, Richard Fernandes, Tamar Schildkraut, Riddhi Joshi, Lyman Lin, Sara Vorgin, Amirah Etchegaray, Aathavan Shanmuga Anandan, George Tambakis, Moshe Loebenstein, Yoon-Kyo An, Jakob Begun, Emily K Wright

**Affiliations:** Department of Gastroenterology, St Vincent’s Hospital, Melbourne, Australia; Department of Gastroenterology, Mater Hospital, Brisbane, Australia; Mater Research Institute, University of Queensland, South Brisbane, Australia; Department of Gastroenterology, Mater Hospital, Brisbane, Australia; Mater Research Institute, University of Queensland, South Brisbane, Australia; Department of Gastroenterology, St Vincent’s Hospital, Melbourne, Australia; Department of Gastroenterology, St Vincent’s Hospital, Melbourne, Australia; Department of Gastroenterology, St Vincent’s Hospital, Melbourne, Australia; Department of Medicine, University of Melbourne, Melbourne, Australia; Department of Gastroenterology, Mater Hospital, Brisbane, Australia; Department of Gastroenterology, Mater Hospital, Brisbane, Australia; Department of Gastroenterology, St Vincent’s Hospital, Melbourne, Australia; Department of Gastroenterology, St Vincent’s Hospital, Melbourne, Australia; Department of Gastroenterology, Mater Hospital, Brisbane, Australia; Mater Research Institute, University of Queensland, South Brisbane, Australia; Department of Gastroenterology, Mater Hospital, Brisbane, Australia; Mater Research Institute, University of Queensland, South Brisbane, Australia; Department of Gastroenterology, St Vincent’s Hospital, Melbourne, Australia; Department of Medicine, University of Melbourne, Melbourne, Australia

**Keywords:** Crohn’s disease, tumor necrosis factor, infliximab, adalimumab, therapeutic dose escalation, proactive

## Abstract

**Background:**

Antitumor necrosis factor (TNF) dose escalation is performed to improve therapeutic response and optimize outcomes in patients with Crohn’s disease (CD). We aimed to describe the durability of anti-TNF therapy in patients with CD receiving escalated anti-TNF therapy, along with the overall durability of anti-TNF treatment between patients managed with a proactive versus reactive therapeutic drug monitoring (TDM) approach.

**Methods:**

We undertook a retrospective multicentre cohort study. One center practiced proactive TDM with a weekly virtual TDM clinic, while the other practiced reactive TDM. Patients receiving escalated infliximab or adalimumab therapy for CD from January 2015 to April 2022 were included. Durability was defined as the time from biologic start to cessation for treatment failure.

**Results:**

239 patients (45% female, median age 39) meeting criteria for inclusion were identified; 165 patients were included in the proactive TDM cohort and 74 in the reactive TDM cohort.

Anti-TNF naïve patients had significantly higher durability of therapy when compared with the anti-TNF exposed patients for both overall durability (*P* = .045) and durability postescalation (*P* = .017). The proactive TDM cohort had significantly higher durability when compared with the reactive cohort for both overall durability (*P* = .001) and durability postescalation (*P* = .002).

**Conclusions:**

This multicentre, retrospective cohort study illustrates the importance of dose escalation as a therapeutic strategy in IBD care. The durability of anti-TNF therapy is superior in anti-TNF naïve compared to exposed patients and can be improved further by proactive TDM to guide dose optimization.

## Graphical Abstract

**Graphical Abstract:**
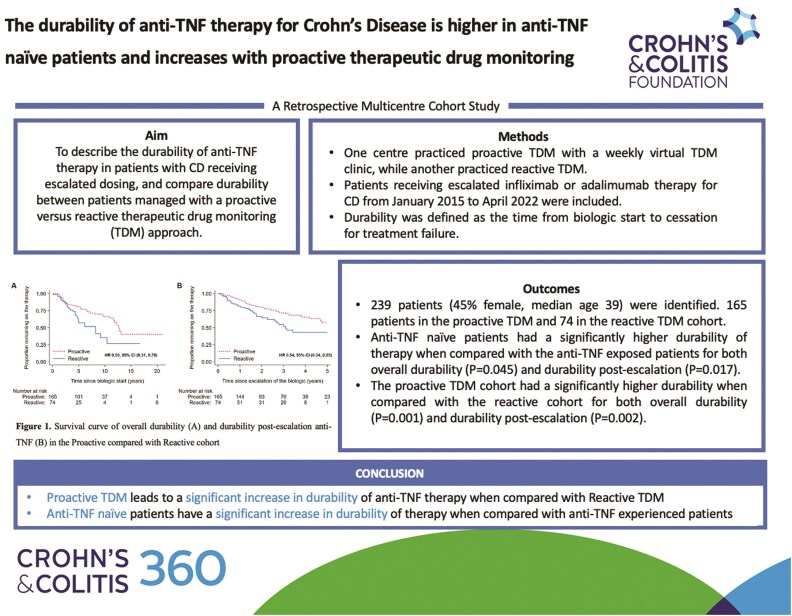

## Introduction

Crohn’s disease (CD) is a chronic, progressive, inflammatory condition causing transmural inflammation of the gastrointestinal tract.^1^ With time, complications including stricture and fistula formation can occur affecting up to 30% of patients.^2,3^ Symptoms can be debilitating and lead to significant physical and psychological disability for patients and increased health care costs.^4^

Current guidelines recommend the early use of immunomodulator therapy with prompt escalation to advanced drug therapy (ADT) for moderate to severe disease.^5^ Most recently the commencement of Infliximab with an immunomodulator at the time of diagnosis has been shown to have superior outcomes compared with conventional step-up therapy highlighting the ongoing importance of anti-TNF therapy in the treatment landscape.^6^ While we have seen an increase in ADT availability for inflammatory bowel disease (IBD) in the past 5 years, there are relatively few new approved therapies for CD, as opposed to Ulcerative Colitis (UC). Patients with Crohn’s disease are also more likely to be treated with ADT earlier in their treatment journey.^7^ Given the relatively young age at diagnosis, patients are likely to require treatment with ADT’s over decades. As such, it is vital that patients are able to achieve maximum therapeutic efficacy and durability from the limited available ADT’s before switching to another agent.

The antitumor necrosis factor (TNF) medications adalimumab and infliximab were the first approved ADTs for the treatment of CD and remain important therapies in our drug armamentarium. Tumor necrosis factor inhibitors are efficacious for moderate to severe luminal and perianal CD, and have been shown to prevent postoperative disease recurrence and improve CD strictures.^8–11^ However, due to the immunogenic nature of these therapies and the development of antitreatment antibodies, loss of response to these drugs remains a major clinical challenge.^12^ To reduce this risk, TNF inhibitors can be combined with an immunomodulator when tolerated, such as a thiopurine, or methotrexate although there are additional risks associated with combination therapy that need to be considered.^13^ Loss of response to anti-TNF therapy can be attributed to either pharmacodynamic failure (high circulating drug levels but loss of clinical efficacy), pharmacokinetic failure (low circulating drug levels and associated loss of therapeutic clinical effect) or immunogenic failure (the presence of antibodies that neutralize circulating drug). In pharmacokinetic failure, therapeutic dose escalation, either by increasing the drug dose or shortening the interval between successive doses, has been shown to increase drug levels and “re-capture” response.^14^ By contrast, only in certain scenarios can pharmacodynamic failure be addressed by dose escalation. In perianal fistulising CD, higher infliximab drug levels have been shown to improve rates of fistula healing, and dose escalation to achieve infliximab levels greater than 10.1 mcg/mL may result in significant benefit for some patients.^15^ Treatment targets for anti-TNF drugs continue to evolve and are influenced by disease phenotype and severity. Dose escalation in response to therapeutic drug monitoring (TDM) can be initiated when drug levels are low in the setting of loss of clinical response (reactive TDM) or when drug levels are low without evidence of loss of clinical response (proactive TDM). It has been proposed that proactive TDM may increase the persistence of anti-TNF therapy, prevent loss of response, reduce complications of active IBD, and decrease overall healthcare utilization from these patients.^16^

Retrospective data and metanalysis suggest a proactive TDM approach is associated with a reduction in treatment failure, hospitalization, need for IBD-related surgery and may increase drug durability.^17,18^ Results of controlled studies, however, have failed to consistently illustrate the benefits of proactive TDM over a reactive approach.^19–21^ No prospective studies have compared anti-TNF treatment durability with respect to TDM and dose optimization approaches.

This study aimed to compare the real-world durability of anti-TNF therapy in patients with CD, comparing those monitored as part of a proactive versus reactive treatment strategy at 2 different tertiary centers in Australia.

## Methods

### Study Design

We performed a retrospective cohort study to assess durability of therapy among patients with CD on escalated anti-TNF inhibitor therapy. The study included patients who commenced dose-escalated anti-TNF therapy for CD between September 2012 and March 2023 from 2 tertiary Australian IBD centers, with one site practicing proactive dose escalation with a weekly virtual biological clinic and the other practicing reactive dose escalation. Inclusion criteria consisted of men and women aged 18 and above with a CD diagnosis of at least 3 months. Patients must have completed infliximab or adalimumab induction therapy (3 doses of infliximab dosed at week 0, 2, and 6 or 2 doses of adalimumab dosed at 160 mg and 80 mg spaced 2 weeks apart), plus an escalation in dosing beyond the standard schedule (5 mg/kg Q8W for infliximab and 40 mg s/c fortnightly for adalimumab) to be eligible for inclusion. The decision to include only patients with escalated dosing only was made to exclude cases of primary nonresponse to anti-TNF who cease therapy prior to undergoing TDM. Patients with indeterminate colitis, UC, prior colectomy including patients with an ileo-anal pouch and patients who were pregnant or breastfeeding were excluded as were those with a recent venous thromboembolism or solid organ malignancy. Ethics approval for this research project was granted by St Vincent’s and Mater Human Research Ethics Committee’s.

Proactive dose escalation was largely protocol driven although exceptions could be made by the lead IBD clinician on a case-by-case basis. All drug level results and anti-TNF dose changes were discussed a weekly multidisciplinary meeting comprising an IBD clinician/s, IBD fellow, IBD nurse consultant and/or IBD Nurse Practitioner and pharmacist. Patients were reviewed 3 months after anti-TNF start, dose escalation or de-escalation and at 6 monthly intervals otherwise with their available clinical progress, drug levels, and biomarkers being reviewed. Anti-TNF drug levels were checked prior to each of the above timepoints. In patients in remission, dose escalation was undertaken to ensure infliximab or adalimumab level of >3μg/mL. In patients with clinically or biochemically active luminal disease, dose escalation was undertaken to ensure infliximab level of >5μg/mL or adalimumab level of >7μg/mL. In patients with active perianal disease, dose escalation was undertaken to ensure infliximab or adalimumab levels >10μg/mL. Patients with low (<1μg/mL) or undetectable infliximab or adalimumab levels were tested for the presence of antibodies, and if present current anti-TNF therapy was ceased and switched to an alternate appropriate therapy.

Reactive dose escalation represents current standard of care. Drug levels were completed 3 months after anti-TNF start, dose escalation or de-escalation and at 6 monthly intervals otherwise, but dosing escalation was not protocolized and did not occur on level alone. Anti-TNF dose escalation was based on clinical or biochemically active disease, with drug levels reviewed at physician’s discretion. The decision to optimize therapy by increasing drug dose or shortening interval between doses was clinician-dependent.

### Outcomes and Definitions

The primary outcome was overall durability of anti-TNF therapy. In keeping with prior studies, durability was defined as the time from initiation of therapy to cessation for treatment failure.^22,23^ Treatment failure was defined as drug discontinuation for secondary loss of response or need for IBD-related surgery. Secondary loss of response was defined as documented clinical response to induction therapy, with subsequent loss of response with clinically or biochemically active disease during maintenance therapy. Patients where drug was discontinued because of adverse events or because of the achievement of remission were excluded.

Secondary outcomes included: (1) Durability of therapy from time of dose escalation, (2) Durability of anti-TNF naïve vs anti-TNF exposed patients, and (3) Factors associated with durability of therapy.

Clinically active disease was defined as active clinical symptoms (based on PRO-2 criteria) plus at least one objective marker of disease activity (raised c-reactive protein [CRP], fecal calprotection [FCP] or abnormal intestinal ultrasound, magnetic resonance enterography or endoscopy). Biochemically active disease was defined as FCP > 250 mcg/g or CRP > 10 mg/L.

### Demographic and Clinical Data

Demographic and clinical data were collected by retrospective chart review. The following demographic data was collected: age, sex, smoking history, age at diagnosis, duration of disease, location of disease, behavior of disease including perianal involvement, surgical history, presence of extra-intestinal manifestations, prior and current medication including previous biologic therapy. All concomitant medical therapies continued during the period of follow-up were documented, including use of corticosteroid and cumulative corticosteroid dose.

Clinical and biochemical data were collected at set timepoints: at baseline (prior to commencement of anti-TNF), at time of initial escalation (±14 days), at time of subsequent escalations (±14 days) and at time of treatment failure. Clinical data included reason for dose escalation and disease activity (measured by physician global assessment). Biochemical data included anti-TNF drug levels (by enzyme-linked immunoassay [ELISA]), presence of antidrug antibodies (by ELISA), thiopurine metabolites if applicable, c-reactive protein (CRP: normal 0-10 mg/L), albumin, and fecal calprotectin (FCP: normal 0-250 mcg/g).

### Statistical Analysis

Continuous variables were summarized using medians and interquartile ranges (IQRs), while frequency and percentage were presented as categorical variables. Participants’ characteristics were compared between the groups using rank sum test (continuous variables) and Fisher’s Exact test (categorical variables). Durability of biologic (overall and escalated therapy) was evaluated using Cox proportional hazards regression and displayed using Kaplan Meier curves. Variables with *P* < .20 on univariable analysis were included in the multivariable model. Results are presented as hazard ratios (HR) with 95% confidence intervals (CI). All analysis was performed using Stata 18 (StataCorp LLC, College Station, TX).

## Results

A total of 239 patients meeting criteria for inclusion were identified. The clinical and demographic characteristics of the cohort are summarized in Table 1.

**Table 1.** Patient demographics stratified by TDM.

Demographics, *n* (%)	Overall Cohort (*n* = 239)	Proactive TDM (*n* = 165)	Reactive TDM (*n* = 74)	*P*-value
Female	108 (45)	76 (46)	32 (43)	.78
Median age at time of induction, years (IQR)	39 (30-49)	41 (33-50)	32 (22-45)	<.01
Median weight at time of escalation, kg (IQR)	79 (63-92)	80 (66-93)	75 (62-88)	.10
Disease duration, years (IQR)	4.5 (1.2-8.7)	5.8 (1.2-9.3)	3.0 (1-7.8)	.11
Montreal classification—age at onset				
A1 <16 years	55 (23%)	32 (19)	23 (31)	.11
A2—17-40 years	158 (66%)	114 (69)	44 (59)	.08
A3 >40 years	26 (11%)	19 (12)	7 (10)	.31
Montreal classification—disease location				
L1—ileal	36 (15)	17 (10)	19 (26)	.16
L2—colonic	70 (29)	57 (35)	13 (18)	.08
L3—ileocolonic	130 (55)	88 (53)	42 (57)	.31
L4—isolated upper GI	3 (1)	3 (2)	0 (0)	.07
Montreal classification—disease behavior				
B1—uncomplicated	84 (35)	44 (27)	40 (54)	<.01
B2—stricturing	82 (34)	61 (37)	21 (28)	.15
B3—penetrating	73 (31)	60 (36)	13 (18)	<.01
Perianal disease	132 (55)	92 (56)	40 (54)	.89
Prior surgery	115 (48)	87 (53)	28 (38)	.04
Current smoker	48 (20)	40 (24)	8 (11)	.02
Tumor Necrosis Factor Naïve	173 (72)	111 (67)	62 (84)	<.01
Infliximab escalation	126 (53)	88 (53)	38 (51)	.78
Adalimumab escalation	113 (47)	77 (47)	36 (49)	.78
Concurrent Immunomodulator use	152 (64)	111 (67)	41 (55)	.08
Anti-TNF drug level availability at time of escalation	182 (76)	128 (78)	54 (73)	.17
Anti-TNF drug level at escalation, median (IQR)	3.3 (1.7-5.1)	3.1 (1.7-5.0)	3.6 (1.8-5.2)	.08

Patients had a median age of 39 years (IQR 30-49), 45% were female, the median duration of disease was 4.5 (IQR 1-9) years, and 20% were active smokers. 64% were receiving combination therapy with an immunomodulator for the duration of their follow-up, with median follow-up from initiation of anti-TNF of 5.4 years (IQR 2.6-8.5 years). At the end of follow-up 67% of patients remained on escalated therapy.

### Proactive vs Reactive TDM

At study commencement, 52% of all patients treated with adalimumab and 47% of all patients treated with infliximab were receiving escalated dosing at the center practicing proactive TDM, and were included in the proactive TDM cohort. In comparison, 35% of all patients treated with adalimumab and 33% of all patients treated with infliximab were receiving escalated dosing at the center practicing reactive TDM, and were included in the reactive TDM cohort. The center practicing proactive TDM had a significantly higher proportion of patients receiving escalated dosing of both infliximab (52% vs 35%, *P* = .02) and adalimumab (47% vs 33%, *P* = .04) at baseline.

About 165 patients were included in the proactive TDM cohort, with 74 included in the reactive TDM cohort for analysis. The clinical and demographic characteristics of these cohorts are summarized in Table 1. Patients across both cohorts shared some similarities; however, those in the proactive TDM cohort tended to be older, were more likely to be smokers, have a colonic and/or penetrating disease phenotype, were more likely to have undergone prior-CD related surgery and more likely to have had previous exposure to anti-TNF therapy.

Anti-TNF drug levels were available for 78% of patients in the proactive TDM cohort, compared with 73% in the reactive cohort at time of escalation of anti-TNF and time of cessation or end of follow-up. Given the real-world nature of this study, those without available anti-TNF drug levels were still included for analysis.

The proactive TDM cohort had a median infliximab drug level of 3.0 μg/mL at time of escalation, compared to 3.5 μg/mL (*P* = .17) in the reactive TDM cohort. The proactive TDM cohort had a median infliximab drug level of 11.2 μg/mL at time of cessation and 12.5 μg/mL at end of follow-up, compared to 8.2 μg/mL (*P* = .02) at the time of cessation and 11.4 μg/mL (*P* = .23) at the end of follow-up in the reactive TDM cohort. Anti-infliximab antibodies were present in 13% of patients receiving infliximab in the proactive TDM cohort and present in 74% of those with low anti-infliximab drug levels during the follow-up period. Anti-infliximab antibodies were present in 16% of patients receiving infliximab in the reactive TDM cohort and present in 62% of those with low infliximab drug levels during the follow-up period.

The proactive TDM cohort had a median adalimumab drug level of 2.8 μg/mL at time of escalation, compared to 3.7 μg/mL (*P* = .09) in the reactive TDM cohort. The proactive TDM cohort had a median adalimumab drug level of 12.5 μg/mL at time of cessation and 11.2 μg/mL at end of follow-up, compared to 8.2 μg/mL (*P* = .01) at the time of cessation and 10.4 μg/mL (*P* = .19) at the end of follow-up in the reactive TDM cohort. Antiadalimumab antibodies were present in 11% of patients receiving adalimumab in the proactive TDM cohort, and present in 63% of those with low antiadalimumab drug levels during the follow-up period. Antiadalimumab antibodies were present in 16% of patients receiving adalimumab in the reactive TDM cohort, and present in 54% of those with low adalimumab drug levels during the follow-up period.

**Figure 1.**
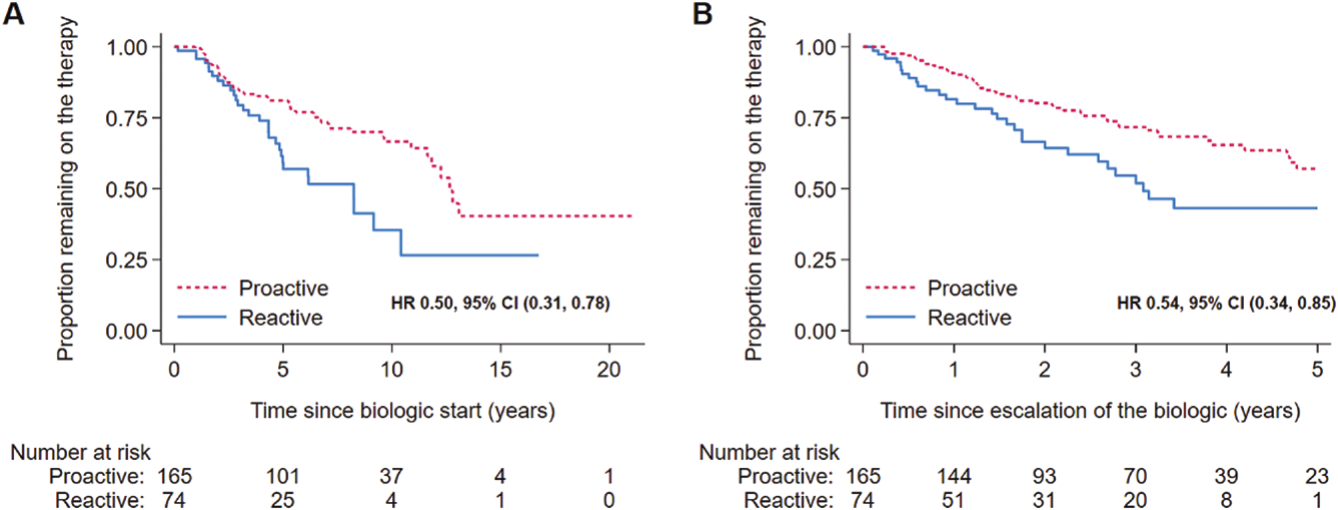
Survival curve of overall durability (A) and durability postescalation anti-TNF (B) in the proactive compared with reactive cohort.

At the end of follow-up more patients in the proactive TDM cohort compared to the reactive cohort remained on escalated anti-TNF therapy (70% [median duration 6.5 years] compared to 60% [median duration 4.3 years], respectively, *P* = .03). On univariable analysis (Table 3), reactive TDM was associated with a significantly lower overall durability of therapy compared with proactive TDM (HR 0.50, 95% CI (0.31, 0.78), *P* = .003; Figure 1A). For durability of therapy after initial dose escalation (Table 3), a proactive TDM approach was also associated with a significantly increased durability when compared with reactive TDM (HR 0.54 (0.34, 0.85), *P* = .008; Figure 1B). These findings remained significant when corrected for confounders, including disease location, disease behavior, perianal disease, prior anti-TNF exposure, smoking status, prior surgery, and concurrent immunomodulator use (Table 4).

**Table 3.** Univariable analysis comparing durability post exposure and durability postescalation.

	Durability postexposure		Durability postescalation	
	HR (95% CI)	*P*-value	HR (95% CI)	*P*-value
Proactive vs reactive	0.50 (0.31, 0.78)	.003	0.54 (0.34, 0.85)	.008
Male vs Female	1.18 (0.76, 1.83)	.474	1.12 (0.72, 1.74)	.611
Age at time of induction	0.99 (0.98, 1.01)	.396	0.99 (0.98, 1.01)	.328
Weight at time of escalation	0.99 (0.99, 1.01)	.845	1.00 (0.99, 1.01)	.620
Disease duration	0.99 (0.96, 1.01)	.302	0.99 (0.96, 1.01)	.268
Montreal classification—age at onset				
A1 < 16 years	Ref		Ref	
A2 – 17-40 years	0.97 (0.58, 1.62)	.904	0.95 (0.57, 1.59)	.837
A3 > 40 years	1.09 (0.46, 2.58)	.853	0.78 (0.33, 1.84)	.564
Montreal classification—disease location				
L1—ileal	Ref		Ref	
L2—colonic	0.54 (0.28, 1.04)	.066	0.65 (0.33, 1.25)	.192
L3—ileocolonic	0.55 (0.30, 0.99)	.046	0.63 (0.35, 1.13)	.122
L4—isolated upper GI	0.32 (0.04, 2.45)	.273	0.53 (0.07, 4.03)	.540
Montreal classification—disease behavior				
B1—uncomplicated	Ref		Ref	
B2—stricturing	0.88 (0.51, 1.51)	.643	0.80 (0.46, 1.37)	.409
B3—penetrating	0.96 (0.56, 1.62)	.867	0.92 (0.54, 1.57)	.762
Perianal disease	0.50 (0.32, 0.79)	.003	0.45 (0.29, 0.70)	<.001
Prior surgery	1.20 (0.77, 1.86)	.423	1.25 (0.80, 1.95)	.319
Current smoker	1.19 (0.69, 2.07)	.529	1.14 (0.66, 1.97)	.639
Tumor Necrosis Factor Naïve	0.76 (0.48, 1.21)	.245	0.74 (0.47, 1.17)	.199
Infliximab vs adalimumab escalation	0.81 (0.52, 1.26)	.355	0.87 (0.56, 1.35)	.537
Concurrent immunomodulator use	0.73 (0.47, 1.15)	.173	0.72 (0.46, 1.12)	.143

**Table 4.** Multivariable analysis comparing durability postexposure and durability postescalation.

	HR (95% CI)	*P*-value	HR (95% CI)	*P*-value
Proactive vs reactive	0.42 (0.25, 0.70)	.001	0.44 (0.26, 0.74)	.002
Montreal classification—disease location				
L1—ileal	Ref		Ref	
L2—colonic	0.74 (0.37, 1.46)	.380	1.01 (0.51, 2.00)	.979
L3—ileocolonic	0.60 (0.32, 1.11)	.101	0.77 (0.41, 1.42)	.400
L4—isolated upper GI	0.48 (0.06, 3.84)	.491	1.18 (0.15, 9.41)	.875
Montreal classification—disease behavior				
B1—uncomplicated	Ref		Ref	
B2—stricturing	1.34 (0.73, 2.48)	.348	1.10 (0.61, 1.97)	.759
B3—penetrating	2.10 (1.10, 3.98)	.024	1.97 (1.05, 3.68)	.034
Perianal disease	0.40 (0.24, 0.67)	<.001	0.35 (0.21, 0.58)	<.001
Tumor Necrosis Factor Naïve	0.63 (0.39, 1.01)	.045	0.54 (0.33, 0.90)	.017
Concurrent immunomodulator use	0.75 (0.48, 1.18)	.212	0.71 (0.45, 1.14)	.155

### Anti-TNF-Naïve vs Anti-TNF-Exposed Patients

About 173 patients were included in the anti-TNF naïve cohort, with 66 included in the anti-TNF exposed cohort for analysis. The clinical and demographic characteristics of these cohorts are summarized in Table 2. Patient cohorts were well balanced although there was a significantly higher proportions of female patients and those on concurrent immunomodulator therapy in the anti-TNF exposed compared to the anti-TNF naïve cohort.

**Table 2.** Patient demographics stratified by anti-TNF exposure.

Demographics, *n* (%)	Anti-TNF Naive (*n* = 173)	Anti-TNF Exposed (*n* = 66)	*P*-value
Female	71 (41)	37 (56)	.04
Median age at time of induction, years (IQR)	37 (29-50)	41 (30-49)	.56
Median weight at time of escalation, kg (IQR)	79 (65-91)	79 (62-98)	.99
Disease duration, years (IQR)	4.0 (1.1-8.4)	5.3 (1.3-9.0)	.38
Montreal classification—age at onset			
A1 < 16 years	39 (23)	16 (24)	.39
A2—17-40 years	114 (66)	44 (67)	.46
A3 > 40 years	20 (11)	6 (9)	.43
Montreal classification—disease location			
L1—ileal	27 (16)	9 (14)	.37
L2—colonic	51 (29)	19 (29)	.49
L3—ileocolonic	92 (53)	38 (57)	.24
L4—isolated upper GI	3 (2)	0 (0)	.15
Montreal classification—disease behavior			
B1—uncomplicated	64 (37)	20 (30)	.25
B2—stricturing	59 (34)	23 (35)	.42
B3—penetrating	50 (29)	23 (35)	.18
Perianal disease	94 (54)	38 (58)	.67
Prior surgery	81 (47)	34 (52)	.56
Current smoker	38 (22)	10 (15)	.28
Proactive TDM	111 (64)	54 (82)	<.01
Infliximab escalation	95 (55)	31 (47)	.31
Adalimumab escalation	78 (45)	35 (53)	.31
Concurrent immunomodulator use	102 (59)	50 (76)	.02

**Figure 2.**
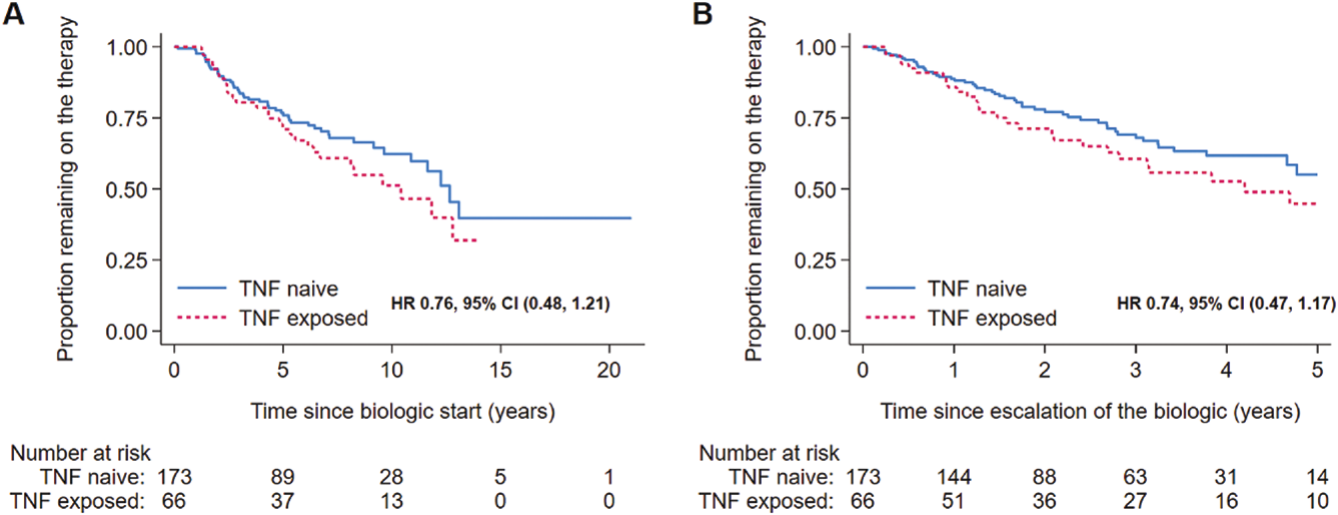
Survival curve of overall durability (A) and durability postescalation anti-TNF (B) in the anti-TNF naïve vs anti-TNF exposed cohort.

At the end of follow-up more patients in the anti-TNF naïve group compared to the anti-TNF exposed group remained on escalated anti-TNF therapy (69% [median duration 5.2 years] vs 58% [median duration 6.1 years], respectively, *P* =< .01). On univariate analysis (Table 3), the anti-TNF naïve cohort showed improved overall durability as well as durability of therapy postescalation, however, neither effect was statistically significant (Table 3; Figure 2). When, however, adjusted for other confounders (disease location and behavior, perianal disease, proactive treatment, and concurrent modulator use), the observed effect increased to reach significance (Table 4).

On multivariate analysis, perianal disease was associated with superior durability of anti-TNF treatment, while penetrating disease behavior is associated with inferior durability. Concurrent immunomodulator use was not associated with a significant improvement durability over anti-TNF monotherapy (Table 4).

## Discussion and Conclusions

Despite the expanding therapeutic armamentarium for Crohn’s disease, anti-TNF therapies are an important part of treatment for many patients. Loss of response to anti-TNF therapies is a common and challenging clinical problem. Any strategy that may improve the durability of anti-TNF therapy presents an important opportunity to improve patient outcomes. Here, we present a large multicentre, retrospective cohort study demonstrating that a proactive TDM strategy for anti-TNF dose escalation is associated with higher durability of anti-TNF therapy in patients undergoing dose escalation. We also found that anti-TNF naïve patients have higher durability of therapy compared to anti-TNF exposed. On multivariate analysis, anti-TNF exposed patients managed with reactive TDM had significantly reduced durability compared with all other groups (anti-TNF naïve with reactive or proactive TDM, plus anti-TNF exposed with proactive TDM).

There were some significant demographic differences seen between the proactive and reactive TDM groups. When compared to the reactive cohort, the proactive cohort were older (41 vs 32 years), had a longer duration of disease (5.9 vs 4.2 years), were more likely to smoke (24% to 11%), and more likely to be anti-TNF experienced (33% vs 16%). They were also more likely to have fistulising disease (36% vs 18%) and have had prior surgery (55% vs 38%) compared to those in the reactive cohort. This suggests the proactive TDM cohort had a more aggressive or refractory disease phenotype at baseline. In this light, the benefit of proactive TDM in terms of durability may be underestimated in this study.

Of note, we saw increased durability of anti-TNF therapy on multivariable analysis in patients with perianal disease, compared to those without. The opposite was seen in patients with internal penetrating disease, with a significantly shorter durability of anti-TNF therapy for this cohort. These differences may be explained by a lack of available alternative agents with proven efficacy for perianal disease, resulting in clinicians persisting with therapy longer than they would if alternative therapies were available. Alternatively, the necessity for early surgical management for patients with internal stricturing/penetrating disease could result in earlier referral for surgery and cessation of anti-TNF therapy.

Interestingly, this study showed no increase in anti-TNF durability with concurrent immunomodulator use for either cohort. While randomized prospective studies have shown significant benefit in the form of corticosteroid-free clinical remission and increased anti-TNF levels for patients using combination anti-TNF and immunomodulator for CD,^24,25^ a number of large real-world studies have failed to show significant benefit in any measured outcome.^26,27^ Whether this relates to the inherent bias in retrospective real-world studies or represents a true lack of effect of combination therapy is unclear.

Anti-TNF drug levels were not significantly different between the proactive and reactive cohorts at the time of dose escalation, and drug levels were higher after dose escalation in both reactive and proactive TDM cohorts. Proactive TDM did result in significantly higher anti-TNF levels in those discontinuing therapy compared to those in the reactive cohort (11.2 μg/mL vs 8.2 μg/mL, *P* = .02), but there was no significant difference in anti-TNF drug level in those continuing therapy at the end of follow-up (*P* = .23). The clinical implications of this are unclear, but it may suggest the mechanism for superiority of proactive TDM in terms of durability relates to a reduction in immunogenic loss of response even when drug levels are considered therapeutic.

The durability of medical therapy has often been used as a surrogate for clinical outcomes. Anti-TNF treatment durability has been shown to correlate with reduced IBD-related hospitalizations and surgery in clinical trials.^28–30^ To further confirm this endpoint, Papamichael et al. showed Proactive TDM significantly improved Infliximab durability, with subsequent reduction of immunogenic loss of response, hospitalization, and need for IBD-related surgery.^17^ Assa et al. (PAILOT) showed that proactive TDM in a pediatric population receiving adalimumab for CD resulted in significantly higher rates of corticosteroid-free clinical remission for as long as 72 weeks from initiation of therapy.^21^

In clinical trials, debate remains on the efficacy of proactive compared with reactive TDM. D’Haens et al. (TAILORIX) showed infliximab dose escalation based on a combination of clinical symptoms, biomarkers and drug levels did not improve corticosteroid-free remission compared to routine standard of care.^19^ Vande Castelle et al. (TAXIT) showed that concentration-based dosing of infliximab was not superior to clinically based dosing for achieving remission after 1 year, but it was associated with fewer flares during the course of treatment and some improvement in biochemical inflammation.^20^ Neither study was a true comparison of proactive compared with reactive TDM as described in our study, however, as the TAXIT control group did not consider drug levels at all with dose escalation on a purely clinical basis, and TAILORIX compared TDM to escalation based on symptoms alone regardless of drug levels. Furthermore, the TAXIT study was limited by a short period of follow-up (1 year) used for the primary outcome, given the majority of patients receiving anti-TNF remain on therapy for longer than 12 months. A number of the secondary outcomes, including clinical remission in Crohn’s patients, were positive in TAXIT suggesting potential benefits to a proactive monitoring and treatment approach.

Importantly this study has shown that escalated anti-TNF therapy is common in the Crohn’s disease population. The center practicing proactive TDM had a significantly higher proportion of IBD patients on escalated anti-TNF therapy (at over 50%) when compared to the center practicing reactive TDM. This may have contributed to some the observations made in this study in terms of improved anti-TNF therapy durability at the proactive TDM site. The higher proportion of patients at the proactive TDM site with a more severe disease phenotype or prior anti-TNF failure may also explain some of this difference. Regardless it is increasingly clear that anti-TNF drug dosing can be dynamic with dosing often increased beyond standardized schedules in order to achieve and maintain clinical benefit.

While there is a significant cost associated with escalated dosing of anti-TNF agents, studies have consistently shown that TDM is cost-effective, and in some settings cost-saving.^31^ The increasing availability of more affordable biosimilar medications will further contribute to this benefit.^32^ What remains unclear is the cost-effectiveness of proactive compared to reactive TDM. In our cohort a significantly higher proportion of patients with CD in the center practicing proactive TDM required escalated therapy compared to the reactive center, and while we did not assess cost in this analysis the cost of medication alone would be correspondingly higher. In a simulated cohort, proactive TDM proved similar in terms of cost-benefit.^33^

Strengths of this study include a large patient cohort (239 patients) with extended follow-up available (median 4.5 years). This extended length of follow-up distinguishes this study from the previously mentioned clinical trials, which focused on outcomes at 1 year. While the Tailorix cohort was followed for a long-term extension study,^34^ the numbers followed beyond 1 year were small at only 95 patients.

Limitations of this study include its retrospective nature and the absence of matched timepoints for clinical data collection.

We have shown that the proactive optimization of anti-TNF therapy may be associated with improved outcomes for patients with Crohn’s disease. Prospective studies are needed to further define strategies to personalize treatment and optimize therapeutic decision-making.

These data support proactive TDM as an effective strategy to improve the durability of anti-TNF-treated patients with Crohn’s disease, regardless of previous anti-TNF drug exposure. It may be that this translates to improved disease control and a reduction in long-term disease complications, although this was not specifically addressed in this study. With the widespread availability of biosimilar anti-TNF agents and the lower cost of these agents worldwide proactive TDM and subsequent dose escalation may well be a cost-effective treatment strategy. The adoption of proactive TDM should now be considered as part of best-practice IBD care as a way to personalize and optimize our current therapies. Further prospective research is required to confirm the clinical benefits and cost-effectiveness of a proactive TDM strategy.

## Data Availability

The data that support the findings of this study are available on request from the corresponding author. The data are not publicly available due to privacy or ethical restrictions.
